# Lived Experiences of Adolescent Living with human immunodeficiency virus in Ghana: A Phenomenology Study

**DOI:** 10.1002/nop2.630

**Published:** 2020-09-23

**Authors:** Abdul‐Razak Doat, Elham Navab, Akram Sadat Sadat Hoseini

**Affiliations:** ^1^ Department of Pediatric Nursing School of Nursing and Midwifery Tehran University of Medical Sciences Tehran Iran; ^2^ Department of Critical Care Nursing School of Nursing and Midwifery Tehran University of Medical Sciences Tehran Iran; ^3^ Department of Pediatric Nursing School of Nursing and Midwifery Research center of Quran Hadith and Medicine Tehran University of Medical sciences Tehran Iran

**Keywords:** adolescent, Ghana, hermeneutic, human immunodeficiency virus, lived experience, phenomenology

## Abstract

**Aim and objectives:**

To explore the meaning of living with HIV positive in Ghanaian adolescents.

**Design:**

Hermeneutic phenomenological approach developed by Van Manen methodology (1990).

**Method:**

A purposive sampling of 12 adolescents living with HIV was recruited. Data were collected between September 2019–January 2020 using semi‐structured interviews and analysed using thematic analysis.

**Results:**

Two main themes emerged: Stigmatization and HIV disclosure and Living with a heavy burden. Seven subthemes were also found. Adolescents living with HIV in Ghana face discrimination, rejection and go through psychological distress such as suicidal thoughts, fear of death and hopelessness.

**Conclusion:**

The problems faced by adolescents living with HIV are a result of the inherent beliefs of the Ghanaian society about HIV. Nurses working with ADLHIV should concentrate on identifying challenges and provide support and care, in addition to their treatment.

## 1 INTRODUCTION

HIV is an important health problem worldwide, and the number of people living with HIV worldwide continued to grow (Alp et al., 2011; Golrokhi et al., 2018). According to the 2018 fact sheet on Global HIV& AIDS statistics, out of the 36.9 million people living with HIV, 35.1 million were adults and 1.8 million children. Adolescence is a dynamic time of life, defined by physical, emotional, cognitive and social transitions. Many of these transitions increase adolescents’ vulnerability to HIV infection while necessitating unique approaches to treatment (Patton et al., 2016). Living with HIV as an adolescent raises the exigency for effective support and guidance to ensure they traverse through this developmental stage (Hodgson et al., 2012). In Africa, the difference between women and men in HIV/AIDS prevalence varies from as low as 0.68% in Liberia to as high as 11.5% in Swaziland. A decomposition analysis showed 84% and 92% of the higher prevalence of HIV/AIDS among women in Uganda and Ghana, respectively. This trend is a result of differences in the distribution of HIV risk factors most especially the age at first sex between women and men (Sia et al., 2016). Interestingly, safer‐sex, family planning and disclosure of HIV status gain prominence during adolescence, but HIV‐positive adolescents fear rejection, stigma and public exposure if they disclose their status to their sexual and romantic partners (Toska et al., 2015). It is therefore imperative that adolescents afflicted with HIV are provided with the needed guidance and assistance as they traverse through the developmental phase and assume responsibility of taking treatment, sexual relationship and their general well‐being.

## 2 BACKGROUND

Ever since Ghana recorded its first HIV case in the year 1986 (Commission, 2008), HIV has since been a matter of national priority due to the high mortality rates among persons afflicted with the disease. The estimated number of people who were living in Ghana with HIV at the end of 2018 was 334,713, the number of new infections stood at 19,931, the annual death due to HIV was 14,181, ART coverage for children (0–14 years) stood at 19.9%, ART coverage for adults (15 + years) was 35.16%, and the HIV prevalence during the same period was 1.69% (COMMISSION, 2019). Most Ghanaians hold the belief that certain illnesses are exclusively attributed to sins against the gods, and these beliefs give room for persons afflicted with HIV to be perceived as immoral (Awusabo‐Asare & Anarfi, 1997; Ohemeng et al.., 2016; Poku et al., 2005). A section of Ghanaians beliefs that anyone who contracts HIV becomes dangerous and must be feared hence such persons should be isolated from society (Clottey‐Sefa, 2001; Mwinituo & Mill, 2006). Ghanaians have a strong negative perception about HIV and have used demeaning words to describe the disease, and these include imminent death, untimely death, disgraceful death, shameful death, long‐suffering death and abominable disease (Ohemeng et al.., 2016; Poku et al., 2005).

Ghanaians have a value system which defines their life. They believe that a person should refrain from sexual intercourse until marriage and will blame people afflicted with HIV for the disease for not adhering to this belief (Jnr, 2013; Ulasi et al., 2009). Studies on adolescent in some secondary schools in Ghana revealed that 25% of the participants reported being sexually active; of this set of students, 64.7% had their first sexual bout at age 16 years; and 55.7% of them did not use a condom during sexual intercourse (Adu‐Mireku, 2003). Acts of this nature predispose most Ghanaian adolescents to contract HIV. Studies show that HIV‐related challenges concerning adolescents are constant; thus it is, therefore, necessary to identify these challenges and further investigate them (Ayres et al., 2004; Li et al., 2010). Even though there is growing interest in researching adolescent HIV in Ghana, research on adolescents living with HIV is mostly concentrated on the epidemiology of sexually acquired HIV infection with significant interest in primary prevention of HIV infection among the adolescent population and little attention is given to their lived experiences. The population of adolescents living with HIV/AIDS consists of those who acquired it perinatally and is emerging as a progressively important and unique population whose experiences need to be known and understood and those who were infected in their adolescence (Jena, 2014). The current pace of research with regard to adolescent HIV experiences is still low in comparison with the needs of adolescents living with HIV. Therefore, a shred of strong evidence is needed to inform creative and targeted solutions that will bridge research gaps, inform policy and improve outcomes for adolescents (Armstrong et al., 2018). It is in light of this that conducting a study on adolescent living with HIV positive, will help bring out the needed knowledge and possible solutions which will help shape government policies that will be adolescent‐friendly and will also be geared towards the management of infected persons and shape preventive measures.

## 3 METHODS

### 3.1 Aim

The study aimed to explore the meaning of living as an adolescent with HIV positive in Ghanaian.

### 3.2 Design

The phenomenological study enables us to explore the meaning of living as an adolescent with HIV positive in Ghana. Van Manen's methodology was adopted in this study. Van Manen chronicles interpretive phenomenological method as the amalgamation of interpretation and describing and believes that they are not separate (Van, ). Van Manen's six research activities for conducting hermeneutic phenomenological research which provides a framework for reflecting and interpreting the experience under study were used for this study. In this study, the phenomenon experienced by adolescents living with HIV was identified through interviews.

### 3.3 Participants and recruitment

Participants aged between 14–19 years old were recruited from the HIV clinic of the hospital. Experienced HIV ambassadors living with the disease and some nurses in the clinic were co‐opted to assist with the recruitment of prospective respondents to the study. The prospective respondents were contacted directly by the co‐opted HIV ambassadors and nurses. The first author introduced himself and explained the goal of the research project to prospective participants before they were enrolled in the study. The participants were eight females and four males living with HIV. Purposive sampling was used to recruit and interview 12 HIV‐infected adolescents. Sampling was done to achieve diversity of sample characteristics such as gender, tribe, religion and age. Participants were enrolled onto the study if they met the inclusion criteria of (a) an adolescent living with HIV/AIDS in the northern region of Ghana and is mentally sound or stable, (b) participants living with HIV who can express him/herself and (c) participants living with HIV willing to take part in the study.

### 3.4 Data collection

The collection of data was conducted by the first author between the months of September 2019–January 2020 at Tamale Teaching Hospital. The hospital serves as a referral hospital for people in the northern region, Upper East region, Upper West region, Savannah region, Northeastern region, Bono east region and neighbouring countries that share boundaries with Ghana like Togo, Burkina Faso and La Cote D’Ivoire. All authors made significant inputs in coming up with an interview schedule drawing from their wealth of personal, research and nursing experience. Interviews were conducted after having established rapport with respondents, and all the interviews were conducted in English. The interviews were conducted at the nurses’ office. Participants could share their stories using a non‐directive opening question. The question was please share your experiences of living as an adolescent with HIV positive. The interview continued with the question: What is the meaning of living as an adolescent with HIV positive in Ghana? What is the experience of living with HIV like? It was then followed with probing questions, such as Can you give an example? Can you explain further? Each interview was audio‐taped, and it lasted for periods between 30–60 min. Data were collected and transcribed verbatim immediately after the interview. Participants choose time and venue for the interview, and this was dependent on where they felt safe and comfortable.

### 3.5 Data analysis

Data analysis was done according to the third to sixth activities of Van Manen's methodology (Van, ). The process started with reflecting on relevant themes both implicit and explicit which characterizes the phenomenon. After interviews were completed, the audio recordings were transcribed into plain text and verbatim immediately after the interview and the audio recordings were listened to several by principal investigator while comparing with transcribed data. The transcribed data were read and re‐read on each of the twelve transcripts while still comparing with audio recording to be familiar with the data and to avoid errors. Thematic analysis was done with gathered data in other to arrive at the essential meaning of the experience. Van Manen (1997) champions the use of three approaches to identifying themes in a phenomenological study: the holistic approach, the selective approach and the detailed or line‐by‐line approach. The principal investigator used all three approaches for this study to arrive at in‐depth analysis and interpretation and a deeper understanding of the lived experience of adolescents afflicted with HIV and what it means to live with the disease. In the holistic approach, researchers view the text in its entirety to “capture its meanings” (Van Manen, 1997). A selective approach was employed for the exploration of themes. Each transcript was read and a brief written. As principal investigator was reading and re‐reading the transcribed interviews, he was looking out for patterns in the experiences of respondents that answer the research question. Having immersed himself into the data principal investigator became conversant with the gathered data and started putting bits and pieces together in other to arrive at an essential meaning of the experiences. The principal investigator took the initiative to look for words and expressions that were recurrent throughout the transcripts and were essential to the goal of the study. In the selective approach, the individual transcript was read line by line and statements that reflected the research question were identified. Principal investigator started a detailed line by line coding by reading each sentence to enable him to generate a catalog of codes which are concepts used to provide designation to describe what the respondents are saying. Analysis continued until a deep, rich, related and abstract themes were arrived at.

### 3.6 Ethics

The study was approved by the Ethics Committee of Tehran University of Medical Sciences, Tehran, Iran (Code: IR.TUMS.FNM.REC.1398.091). Permission was also granted by the research unit of the hospital to conduct the study. Information on the aim of the study, confidentiality and the voluntary nature of the study was provided to prospective respondents. Written consent for participation and permission to record interviews was obtained from each participant after explaining the purpose of the study to them. Participant's anonymity and confidentiality were ensured by not capturing their names and address. Recorded interviews were kept under lock and key.

### 3.7 Rigour

To achieve the criteria of rigorous research, the study was done in line with the initial study design. To ensure the credibility of the study, external scrutiny was performed with the initial findings when it was presented to a group of experts at a seminar. Principal investigator purposefully recruited participants who met the inclusion criteria and who could provide in‐depth information on their experiences concerning HIV. To achieve the objective of transferability, the principal investigator gave a thorough description of the study setting. A rich and comprehensive account of adolescent's experiences of living with HIV was also outlined. To ensure the dependability of the study, researchers maintained an audit trail by giving a vivid description of the research process from the inception of the study to the reporting of the study findings. Confirmability was ensured by the researcher providing thick, rich, textual and structural descriptions that carry the lived experiences of adolescent HIV.

## 4 RESULTS

Twelve adolescents living with HIV participated in the study, and eight of them were females, while the remaining four were males. Almost all the major socio‐cultural groups in Ghana were represented, including Akan, Gonja, Ewe, Mole‐Dagbani and Gurunis. Of the 12 respondents who took part in the study, 75% of them had acquired HIV horizontally, while the remaining 25% was the vertical transmission. All 12 respondents had initiated ART, and 58.3% of them had been on ART for periods between 2–12 years. With regard to religion, 66.7% of the respondents were Muslims, while the remaining 33.3% where Christians. 75% of the respondents had some level of education, while the remaining 25% had never been to school. Two main themes emerged as findings of this study: Stigmatization and HIV disclosure and Living with a heavy burden. Respondent characteristics are captured in Table 1.

**Table 1:** Participants characteristics

Participants	Age	Ethnicity	Religion	Level of Education	Gender	Region	HIV Transmission	Age at diagnosis	Age at disclosure	Duration on ART
1	15	Gonja	Christian	Junior secondary school	Male	Savannah Region	Vertical	Not sure	12	About 12 years
2	14	Dagomba	Islam	Junior secondary school	Male	Northern Region	Vertical	14	14	10 years
3	16	Dagomba	Islam	Primary school	Female	Northern Region	horizontal	15	15	1 year
4	15	Ewe	Christian	Senior secondary school	Female	Volta Region	Horizontal	14	14	1 year
5	15	Dagomba	Islam	Senior secondary school	Female	Northern Region	Vertical	15	15	8 years
6	19	Talensi	Christian	Primary school	Female	Upper east Region	Horizontal	16	16	3 years
7	19	Akan	Christian	Primary school	Female	Bono Region	Horizontal	18	18	1 year
8	18	Dagomba	Islam	Never been to school	Female	Northern Region	Horizontal	16	16	2 years
9	16	Mamprusi	Islam	Never been to school	Female	Northeastern Region	Horizontal	15	15	1 year
10	19	Mamprusi	Islam	Never been to school	Male	Northeastern Region	Horizontal	15	15	4 years
11	18	Basari	Islam	Primary school	Male	Northern Region	Horizontal	16	16	2 years
12	19	Dagomba	Islam	Secondary school graduate	Female	Northern Region	Horizontal	16	16	3 years
Average age of	16.91									

### 4.1 Stigmatization and HIV disclosure

Participants of the study had concerns about disclosing their status to others, and even if they did, they were very mindful as to whom they should trust such sensitive information. Adolescents living with HIV (ADLHIV) stated that they found themselves trying to balance between moral correctness and protecting their right to privacy. They complained of being systematically stigmatized on several fronts by persons who either knew their HIV status or suspected they were living with HIV. Some also expressed the fact that they suffered some sort of low self‐esteem to the extent that they found it difficult to even show up at the HIV clinic for their antiretroviral treatment. The 12 adolescents who participated in the study shared an in‐depth of their phenomenon. Four subthemes emerged from this study: Stigma, Feeling of guilt and shame, fear of disclosure and Illegal status disclosure.

### 4.2 Stigma

Some participants (ADLHIV) experienced some form of stigma from family members and neighbours who were preview to their predicament. Participants’ experiences are as a result of the Ghanaian societal perception about HIV disease and the fact that they believe it is a form of punishment from god to the afflicted individuals and their families. They see persons afflicted with the disease as persons who had lived a promiscuous life and for that matter will not want to associate with them in any way:

Like the way I could use my mother's things, I can't use them now because my mother will not allow me to use them. When you touch the thing the way she will even talk to you, you will know that is because of the HIV she is talking to you in that manner, this made me feel bad and I thought of committing suicide. (Participant 6)

Already the people living around me know that I am HIV positive as they know I am HIV positive when I go to the toilet (the house toilet) and come out, they have to scrub it before they use it. When I go to the bathhouse and come out, they have to scrub the bathroom before they enter to use it. All these things they did to me made me feel very bad. (Participant 10)

In the area where I live people always point fingers that this boy has HIV. So when am coming to them some of them run away, this makes me very sad. (Participant 11)

### 4.3 Feeling of guilt and shame

All 12 (ADLHIV) who took part in the study suffered some form of guilt and shame. They indicated that the mode of spread of the disease and the societal beliefs about the disease gave rise to their feeling of guilt and shame. They succumbed to societal views of the fact that the disease affects promiscuous people. Whether one is a Christian, Muslim or a traditionalist, they all hold similar beliefs about the fact that some ailments are punishment from god. Most of the study respondents who were in some form of a relationship had not disclosed their HIV status to their partners. When asked of their reason for keeping it secret, they indicated that they feared they will lose their partners if they divulge their status to their partners. They preferred living with the guilt than disclosing their HIV status to their partners:

I have a boyfriend whom I share an intimate relationship with but he does not know of my HIV status. If I wake up early in the morning or anytime I see him I will be feeling very bad for hiding something away from him but I don't know what to do. (Participant 6)

what I experienced is that when people are gathered somewhere and they are sitting down I can't involve myself with them because I fell if I should involve myself with them they will start pointing at me, they will start discussing my issue. (Participant 10)

### 4.4 Fear of disclosure

A greater number of the respondents of the study stated that they feared people will get to know their HIV status and spread the news to others, because of this most of them preferred to keep their status to themselves apart from some few immediate family members who were aware of their status. Some of them indicated that they are very discrete when it comes to the issue of their HIV status and will not even share or disclose their status with blood family relations. Participants’ reasons for taking such a decision are to protect themselves from the harsh reality of society. Here are some quotes from some of the respondents:

Me in my family I do not have anybody who will help me, in terms of…… you know this northern region if you have it, they will consider you as a prostitute, you went and did prostitution and you got your disease and you will come and worry them. Nobody will help me, so if I know they will not help me why should I go and tell, I didn't tell anybody. (Participant 12)

Respondents who choose to disclose their HIV status disclosed to a close family member they trusted:

I didn't tell anybody about my HIV status, it's only my family my dad and my aunt who are aware of my status. I always keep it to myself so none of my friends is aware so am always careful with them but I relate with them.” (Participant 4)

### 4.5 Illegal status disclosure

Of the 12 participants who took part in this study, 25% of them had their status disclosed to others without their permission. They indicated that most of these persons who indulged in these illegal acts were close family relations and friends:

I didn't tell anyone that I have HIV but some people saw me here at the HIV clinic and went and told my aunt and she called me and asked whether I have HIV and I told her I didn't have the disease. So she threatened to bring me to the hospital to do the test. (Participant 3)

When I started feeling unwell, I was sent to the hospital by one of my colleagues whom I was learning how to sew clothes with. She then went and told my boss of my status and they sacked me from work, this made me feel very bad. (Participant 6)

### 4.6 Living with a heavy burden

Adolescents living with HIV shared varying accounts of their experiences when they first got to know they were afflicted with HIV. Their accounts ranged from a sleepless night, suicidal thoughts, aggression, psychosis, depression, grief and hopelessness. Two subthemes emerged from this study: Hopelessness and Fear of death.

### 4.7 Hopelessness

Adolescents living with HIV shared touching experiences about when they first learned of their HIV‐positive status. Some of them felt it was better they killed themselves than to be enslaved by HIV for the rest of their lives. Some thought of stabbing themselves with a knife, taking in poison or use other crude methods to end their lives. Participants’ recounts giving up on life and feeling they will not be able to pursue their dreams:

When the information came that I have HIV, I was always having a knife in my room so there was a day I just took the knife and I wanted to use it on myself and luckily enough my mom just came. I wanted to kill myself so I will just go away, I will just go away from the earth and have peace of mind. (Participant 1)

I thought I will not live in this world again all my dreams have come to an end. I will not achieve anything again. I thought that was the end of me I can't go to school again, I can't do anything again. I felt like I couldn't live again but my mother gave me hope. (Participant 5)

### 4.8 Fear of death

Some respondents of the study recount the sort of fear that gripped them when they learned of their HIV status. Some of the accounts from ADLHIV were to the effect that they feared they will die prematurely form HIV. The premise of their fear is based on stories they have heard from people about HIV and what it does to its victims:

Anytime I will be thinking of that sickness I will be thinking of death because they say when you fall sick with that sickness it will lead you to death. I do not want to die now. (Participant 2)

As they said I was HIV positive I concluded myself as a dead person. I thought that within a week or a month or that year I was going to die for that matter I should not have time to be decorating my room or doing certain things, I was having some fear in me. Fear of death is what I experienced when I was diagnosed HIV positive. (Participant 10)

## 5 DISCUSSION

In this study, ADLHIV shared their experiences with regard to their status disclosure. They expressed fear of being stigmatized if others get to know about their HIV status. Most of the participants expressed fear of being ignored, neglected if their friends or others get to know their HIV status. This is supported by a review study on HIV disclosure in sub‐Saharan Africa where caregivers revealed that the fear of stigmatization is one of the barriers to HIV status disclosure (Doat et al., 2019). In a related study on status disclosure in South Africa, the findings revealed that the level of felt and anticipated stigma is intense and affects all dimensions of living with HIV ⁄AIDS, particularly disclosure and treatment (Gilbert & Walker, 2010).

Illegal status disclose was another finding from the study. This experience is corroborated by the findings of a study conducted in southern India on disclosure of HIV infection. The findings revealed that 24 (35%) out of 68 participants of the study reported that information regarding their HIV status was disclosed without consulting them (Chandra et al., 2003). Noteworthy, findings from a study conducted on stigma in HIV‐positive women revealed that discrediting clues and cues, such as skin lesions and being seen at an HIV clinic, gave women's HIV status away. The findings further revealed that a woman's HIV status was disclosed to others without their permission (Sandelowski et al., 2004).

The study revealed that some respondents had disclosed their status to at least one member of their family. They did this with the conviction that these persons will be able to keep their status secret and not broadcast it to others. This is corroborated by a study conducted on 40 HIV patients in Kampala, which revealed that 95% of the respondents stated disclosing their status to someone, among these persons who disclosed their status 84% disclosed to only family members (Ssali et al., 2010). Another case in point is a study conducted on persons living with HIV in South Africa, where findings of the study revealed that persons living with HIV often disclosed their status first to one trusted family member who is capable of keeping news of the diagnosis secret for a long period of time prior to revealing his status to others (Maman et al., 2014).

The feeling of guilt and shame was experienced by study respondents. They shared their experience of finding it difficult to mingle with people at the early stages of the disease, for fear of being identified as living with HIV. This is corroborated by the study conducted in western Kenya, where guilt was primarily described as a perceived factor influencing women who do not want to test their baby for HIV (Kohler et al., 2014). A study conducted in Singapore revealed that participants felt guilty because their HIV status could potentially cause themselves, their family and extended families to lose respect in society (Ho & Goh, 2017).

25% of participants who took part in the study shared their experience of how they were systematically stigmatized against their own will and had to go through the ordeal thinking of when they will finally take their last breath. The challenges of (ADLHIV) with respect to stigma are corroborated in the findings of a study in South Africa where most participants agreed that people living with AIDS are neglected, ignored and isolated (Meiberg et al., 2008). Noteworthy, stigma on persons living with HIV affects disclosure of HIV status and promotes sexual risk‐taking (Parsons et al., 2004).

Further findings from this study revealed that (7) 58.3% out of the 12 (100%) (ADLHIV) at some point suffered some sort of psychological distress and thought of committing suicide. This finding is supported by the study conducted in Uganda on emotional and behavioural disorders in adolescent living with HIV, and the findings revealed that 51.2% of adolescents living with HIV had significant psychological distress and had attempted suicide (Musisi & Kinyanda, 2009). Another study conducted on Suicidal thoughts and behaviour among South African adolescents living with HIV, 46% experienced one or more symptoms of depression, and 8% reported some level of suicidal thoughts (Casale et al., 2019).

The fear of death was another intriguing finding from this study, ADLHIV expressed fear of dying prematurely. They expressed fear that they might not be able to accomplish their dreams before death catches up with them. These findings are collaborated by the findings of a study conducted on adolescent living with HIV in Kano, and the findings revealed that 54.5% of participants reported being anxious or depressed most of the time for fear of death (Lawan et al., 2015). In another study, the fear of death and dying influenced parents living with HIV’s decision not to disclose their status to their children (Madiba, 2013).

Hopelessness was a major feature in this study with ADLHIV expressing reactions such as difficulties coping and adjusting, loss of interest in the immediate environment and giving up on everything. This is collaborated by the findings of a study conducted in Harare, Zimbabwe, on HIV‐positive adolescents. The findings revealed that young people reported that learning their HIV status resulted in feelings of despair and hopelessness, coupled with a sense of imminent death (Mavhu et al., 2013).

## 6 STUDY LIMITATION

During the study, it became evident that working on a large number of participants will be impossible due to the sheer volume of data uncovered during the research process. Most prospective participants who could have been used for the study fell short of one aspect of the inclusion criteria. They did not know they were living with HIV, though they were coming for regular treatment. Thus, they were exempted from the study.

## 7 CONCLUSION

In this study, HIV disclosure and stigma with its related effects on ADLHIV were observed. The fear of stigma and stigmatization by others made it difficult for adolescents to freely disclose their HIV status to others. Adolescents faced rejection from friends and some family members as they struggle to adjust to the disease. The study also found that adolescents afflicted with HIV experienced some psychological distress in the form of suicidal thoughts, fear of dying prematurely and hopelessness. Nurses working with ADLHIV should, therefore, concentrate on identifying these challenges and provide support and care, in addition to their treatment. Nurses should provide extensive public education on HIV/AIDS, its mode of spread, treatment and prevention. This will improve the knowledge and attitude of Ghanaians towards persons living with HIV. The Ministry of Health should establish a support system that is tailored to meet the needs of ADLHIV at various health facilities where adolescents afflicted with HIV can walk in and access these services. There is a need for further research on the effect of family and peers on the experiences of adolescents living with HIV. The knowledge generated will help in better management and improve the quality of life of adolescents afflicted with HIV.

## CONFLICT OF INTEREST

The authors declare that they have no competing interest.

## AUTHOR CONTRIBUTIONS

All the authors participated in conception and the design. All authors approved the final manuscript. Akram Sadat Sadat Hosseini coordinated the group.

## Data Availability

All data sets from which the conclusion of this manuscript is hinged are available upon a reasonable request to the first author.
